# Spatial patterns of HIV prevalence and service use in East Zimbabwe: implications for future targeting of interventions

**DOI:** 10.7448/IAS.20.1.21409

**Published:** 2017-02-28

**Authors:** Robin Schaefer, Simon Gregson, Albert Takaruza, Rebecca Rhead, Tidings Masoka, Nadine Schur, Sarah-Jane Anderson, Constance Nyamukapa

**Affiliations:** ^a^ Department of Infectious Disease Epidemiology, Imperial College London, Norfolk Place, London, UK; ^b^ Biomedical Research and Training Institute, 10 Seagrave Road, Harare, Zimbabwe

**Keywords:** Spatial heterogeneity, HIV clustering, targeted interventions, equity, Zimbabwe

## Abstract

**Introduction**: Focusing resources for HIV control on geographic areas of greatest need in countries with generalized epidemics has been recommended to increase cost-effectiveness. However, socioeconomic inequalities between areas of high and low prevalence could raise equity concerns and have been largely overlooked. We describe spatial patterns in HIV prevalence in east Zimbabwe and test for inequalities in accessibility and uptake of HIV services prior to the introduction of spatially-targeted programmes.

**Methods**: 8092 participants in an open-cohort study were geo-located to 110 locations. HIV prevalence and HIV testing and counselling (HTC) uptake were mapped with ordinary kriging. Clusters of high or low HIV prevalence were detected with Kulldorff statistics, and the socioeconomic characteristics and sexual risk behaviours of their populations, and levels of local HIV service availability (measured in travel distance) and uptake were compared. Kulldorff statistics were also determined for HTC, antiretroviral therapy (ART), and voluntary medical male circumcision (VMMC) uptake.

**Results**: One large and one small high HIV prevalence cluster (relative risk [RR] = 1.78, 95% confidence interval [CI] = 1.53–2.07; RR = 2.50, 95% CI = 2.08–3.01) and one low-prevalence cluster (RR = 0.70, 95% CI = 0.60–0.82) were detected. The larger high-prevalence cluster was urban with a wealthier population and more high-risk sexual behaviour than outside the cluster. Despite better access to HIV services, there was lower HTC uptake in the high-prevalence cluster (odds ratio [OR] of HTC in past three years: OR = 0.80, 95% CI = 0.66–0.97). The low-prevalence cluster was predominantly rural with a poorer population and longer travel distances to HIV services; however, uptake of HIV services was not reduced.

**Conclusions**: High-prevalence clusters can be identified to which HIV control resources could be targeted. To date, poorer access to HIV services in the poorer low-prevalence areas has not resulted in lower service uptake, whilst there is significantly lower uptake of HTC in the high-prevalence cluster where health service access is better. Given the high levels of risky sexual behaviour and lower uptake of HTC services, targeting high-prevalence clusters may be cost-effective in this setting. If spatial targeting is introduced, inequalities in HIV service uptake may be avoided through mobile service provision for lower prevalence areas.

## Introduction

Geographic variation in HIV prevalence and incidence has been demonstrated even in generalized epidemics in eastern and southern sub-Saharan Africa [1–7]. Currently, there is great interest amongst public health planners in whether this geographic variation could be used to increase the cost-effectiveness of HIV/AIDS interventions by focusing interventions towards areas of highest need [6,8–10]. Increases in cost-effectiveness are needed urgently as UNAIDS estimates that, globally, an additional US$12 billion in HIV/AIDS funding is needed each year to achieve the 90–90–90 targets (90% of HIV-positive people know their status, 90% of these are on treatment, 90% have suppressed viral loads) by 2020 [8]. Furthermore, the potential to improve cost-effectiveness through a focused rather than a uniform national approach has been demonstrated in a modelling study [11].

Several studies, particularly in South Africa [1,2,4], have demonstrated the feasibility of obtaining high-resolution geographic HIV data for a spatial approach to resource allocation but, to date, only a few countries (e.g. South Africa [12] and Mozambique [13]) have implemented an explicitly geographically-focused approach for allocating HIV resources. This is principally because data on the spatial distribution of HIV are often only available for large geographic units [3]. However, this situation is likely to change in the near future with the increasing availability of locally-specific surveillance data from prevention of mother-to-child HIV transmission programmes [14] and improved methods for producing local estimates [7,15].

One serious – but largely neglected – concern with directing treatment and prevention resources to areas of greatest HIV prevalence is the implications for equitable access to health services [16–18]. In particular, populations living outside areas where HIV prevalence is highest may still be subject to considerable epidemics but be poorer and less educated and, therefore, less well-equipped to access HIV services. Especially in rural areas, long travel distances to access HIV services can pose additional challenges as has been shown not only in sub-Saharan Africa [19,20] but also in developed countries (e.g. in the USA [21]). From this standpoint, before implementing geographically-focused interventions, it is important to establish pre-existing patterns of service provision and uptake in areas of high and low HIV prevalence and to be cognisant of any socioeconomic differences between populations living in these areas. Understanding factors associated with geographic variation in HIV prevalence may also allow for the most suitable interventions to be selected and applied in areas of greater and lesser need, but few studies have examined this [4,5].

In this article, we explore factors likely to influence the cost-effectiveness and equity implications of a geographic approach to HIV resource allocation through a case study in east Zimbabwe – a high HIV prevalence country where spatial targeting is currently under consideration [22]. In particular, we aim: (i) to establish whether high or low HIV prevalence clusters exist in selected areas of Manicaland province; (ii) to establish whether clusters of high or low HIV service uptake exist within these areas; (iii) to assess how availability and uptake of HIV services vary between areas of high and lower HIV prevalence; and (iv) to compare socioeconomic and sexual behavioural characteristics of populations living in areas of high and lower HIV prevalence.

## Methods

### Study data

**Figure 1.**
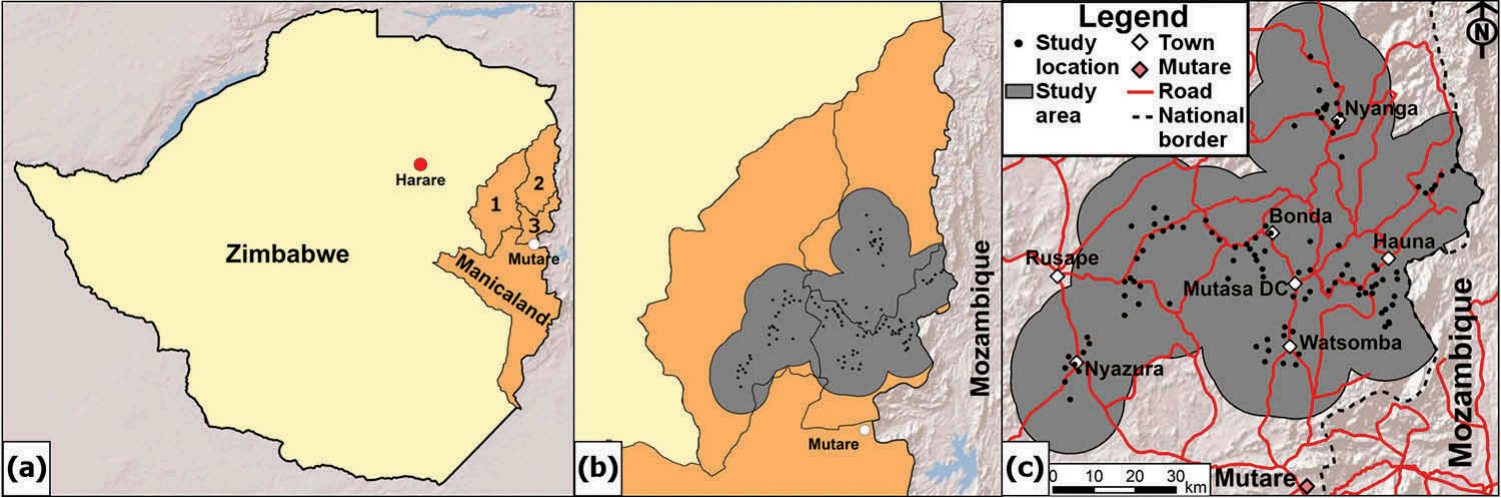
Study districts in Manicaland, Zimbabwe, and study area. (a) Zimbabwe with the three study districts in Manicaland (1: Makoni; 2: Nyanga; 3: Mutasa). (b) The study area (grey) for the interpolation mapping was restricted to the dissolved area of 12.5 km radius circles around each study location (black dots) within the study districts to create a continuous mapping surface. (c) The study area for the interpolation mapping with study locations, larger settlements, the provincial capital (Mutare) and roads indicated.

Data for this study were taken from the Manicaland HIV/STD Prevention Project (Manicaland Project), a longitudinal survey which examines HIV transmission dynamics and its impact in three districts in Manicaland, east Zimbabwe (Figure 1). Since 1998, six rounds of a general-population open-cohort survey have been completed, covering demographic and socioeconomic characteristics, sexual behaviour, knowledge of HIV/AIDS, and availability and uptake of HIV services. Dried blood samples are collected for HIV sero-testing. Written informed consent was gained before enrolment in the survey. Ethical approval for the Manicaland Project was obtained by the Research Council of Zimbabwe and the Imperial College London Research Ethics Committee. Further details of the Manicaland Project are provided elsewhere (see http://www.manicalandhivproject.org/and Gregson et al. [23]).

Here we use data from the sixth round of the survey, conducted between August 2012 and November 2013 in eight sites representing small towns, agricultural estates, roadside trading centres, and subsistence farming villages. Adults aged 15–54 years eligible for detailed interviews and HIV testing were selected from a random sample of two-thirds of households identified in a household census. Eight thousand ninety-two adults participated (61% female) out of 10,410 eligible adults (77.7% participation rate). Geographic positioning system (GPS) data were collected at town- and village-centres of all participating locations, and participants were linked to these 110 locations.

HIV prevalence in each location was calculated from the HIV test results obtained in the survey. Three forms of HIV services were considered – voluntary medical male circumcision (VMMC), HIV testing and counselling (HTC), and antiretroviral treatment (ART). VMMC uptake was based on self-reported medical circumcision amongst males aged 15–29 as the Zimbabwean VMMC programme targets younger males aged 13–29 [22,24]. HTC uptake was calculated as the proportion of all participants reporting having had at least one HIV test in the past three years. ART uptake was calculated as the proportion of adults found to be HIV-positive in this study reporting having ever taken antiretroviral drugs.

### Detection of spatial clusters of HIV prevalence and HIV service uptake

Interpolation maps of HIV prevalence and HTC uptake were created through ordinary kriging with ArcGIS (v.10.2.2, Esri, Redlands, CA, USA) with estimates being directly age-standardized and maps created for both sexes jointly and for females and males separately. The extent of the interpolation area was restricted as seen in Figure 1 and covered 6736 km^2^. The sample sizes for ART (restricted to HIV-positive participants; *n* = 1024) and for VMMC (restricted to males aged 15–29; *n* = 1755) were too small for creating interpolation maps.

Kulldorff spatial scan statistics were used to detect significant clusters of high or low HIV prevalence and uptake of HTC, ART and VMMC [25]. A circular window of various sizes is ranged across the region and, at every location, the number of HIV-positive cases and cases of HTC, ART or VMMC within the circle is compared to the outside. This was implemented in SaTScan (v.9.4.2, Kulldorff, Boston, MA, USA). Only clusters approaching significance (*p* < 0.1) are reported. Analyses were age-adjusted and conducted by sex and for both sexes combined. Sensitivity analyses were conducted by implementing the Kulldorff scan procedure for different definitions of HTC and ART uptake (supplementary material).

Further details of the methods used in the analysis of geographic patterns in HIV prevalence and HIV service uptake are provided in the supplementary material.

### Availability and uptake of HIV services across HIV prevalence clusters

Availability and uptake of VMMC, HTC and ART services in the largest statistically-significant high HIV prevalence cluster (seven locations) and in the significant low HIV prevalence cluster (including 39 locations) were compared separately with availability and uptake in the study areas that did not lie within either the significant high or low HIV prevalence cluster (i.e. areas with intermediate HIV prevalence).

Considering service availability, clusters were characterized in terms of the self-reported distance to the nearest VMMC, HTC and ART services, as well as Euclidean (straight line) distance to the nearest health facility and hospital. Euclidean distance to nearest health facility (including hospital if nearest) and hospital was determined by mapping the 64 known health facilities (including six hospitals) in or close to the study area and measuring the distance from the respondent’s location. No information was available on the actual availability of VMMC, HTC and ART in the health facilities. However, a survey of health facilities in Manicaland found HIV testing and treatment services to be widely available [26], so the nearest clinic is also likely to be a clinic where those services can be accessed, although VMMC services may be less available. Both self-reported and Euclidian distance were used as measures of availability of HIV services as Euclidian distance may not represent the actual travel distance, particularly as road networks were not considered [27]. Reported and Euclidean distances to HIV services were divided along the median into two categories and tests for statistically significant differences in service availability were conducted using logistic regression, adjusting for sex and age. Uptake of HTC (HIV testing past three years), ART (ever treatment) and VMMC (amongst males aged 15–29) were similarly analysed using logistic regressions.

### Differences in socioeconomic and sexual behavioural characteristics of populations living in high and lower HIV prevalence areas

As for HIV services, socioeconomic and sexual behavioural characteristics in the largest statistically-significant high HIV prevalence cluster and in the significant low-prevalence cluster were compared separately with those in the study areas that did not lie within either the significant high or low HIV prevalence cluster. Characteristics examined were sociodemographic (sex, age, marital status and migration), socioeconomic (urban-rural status, education and degree of poverty), and sexual behaviour (age at first sex, number of partners in the last 12 months and condom use at last sexual encounter). Participants who have moved into the study location within the last three years (i.e. since the last survey round) were classified as migrants. Urban-rural status was determined for each location by the average distance to the nearest town, divided into 0–4 (urban), 5–9 (peri-urban) and ≥10 km (rural) categories. For degree of poverty, a wealth index was calculated from various household assets, including sellable (e.g. cars) and non-sellable assets (e.g. water sources) [28], and was divided into terciles. All characteristics were divided into two categories (continuous variables were divided along the median) and tests for statistically significant differences were conducted using logistic regression adjusted for sex and age.

## Results

### Spatial clusters in HIV prevalence

**Figure 2.**
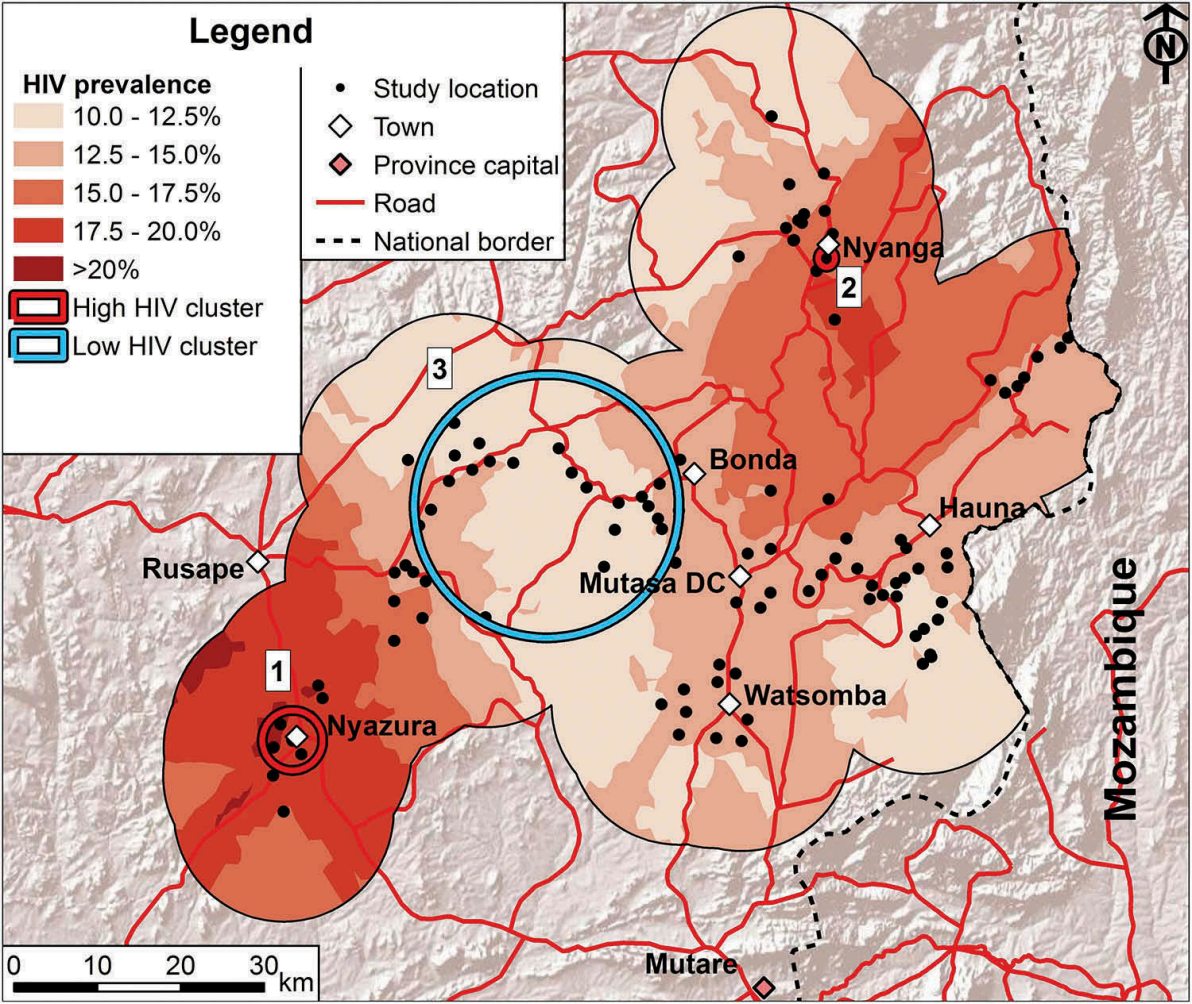
HIV prevalence in the study area. Interpolation of age-standardized HIV prevalence for both sexes combined. Clusters of HIV prevalence are indicated with the numbers corresponding to the numbers in Table 1. For further details on the methodology and the maps for each sex separately see supplementary material.

The interpolation map for HIV prevalence is presented in Figure 2 with the statistically significant high- and low-prevalence clusters superimposed. The HIV prevalence maps by sex show the same general spatial patterns, although female prevalence is generally higher (supplementary material). For both sexes, the more densely-populated urban areas (Nyazura and Nyanga) stand out as having high prevalence whilst other peri-urban areas (e.g. Mutasa DC and Watsomba) do not have a distinctively high prevalence. Significant high HIV prevalence clusters were identified by the Kulldorff spatial scan procedure in the areas around Nyazura (prevalence: 25.7% [*n* = 495] vs. 16.1% in the area outside of the high- and low-prevalence cluster; age-adjusted relative risk [aRR] = 1.78, 95% confidence interval [CI] = 1.53–2.07) and Nyanga (46.5% [*n* = 99] vs. 16.1%; aRR = 2.50, 95% CI = 2.08–3.01) (Table 1A). These clusters were also reflected in the results disaggregated by sex (see supplementary material).

**Table 1.** Clusters of high and low HIV prevalence and VMMC uptake, Manicaland, east Zimbabwe.

****A: HIV prevalence amongst all participants aged 15**–**54****							
	No. of villages	Cluster radius	Cluster population	HIV prevalence	Test for significance		
No.^a^	*N*	(km)	*N*	(%)	aRR^b^	(95% CI)	*p*-value^c^
**High HIV prevalence clusters^d^**							
1	7	3.43	485	25.7	1.78	(1.53–2.07)	<0.001
2	1	0	98	46.0	2.50	(2.08–3.01)	0.003
**Low HIV prevalence clusters^d^**							
3	39	15.05	1376	11.32	0.70	(0.60–0.82)	0.007
							
**B: VMMC uptake amongst males aged 15**–**29**							
	**No. of villages**	**Cluster radius**	**Cluster population**	**VMMC uptake**	**Test for significance**		
**No.^a^**	***N***	**(km)**	***N***	**(%)**	**aRR^b^**	**(95% CI)**	***p*-value^c^**
**High VMMC coverage clusters^e^**							
4	24	7.14	174	10.3	3.26	(1.94–5.72)	0.082
**Low VMMC coverage clusters^e^**							
5	38	10.25	332	0.90	0.19	(0.06–0.61)	0.043

^a^The cluster numbers correspond to the numbers indicated in Figure 2 and 3.

^b^The relative risk and confidence intervals for being HIV-positive (A) or medically circumcised (B) were calculated as the number of participants who were HIV-positive and were medically circumcised, respectively, within the cluster compared to the number not in the cluster (the reference category) adjusted for age delineated into 5-year age groups using the Cochran-Mantel-Haenszel method [29]. Note that the provided HIV prevalence and VMMC uptake statistics are not adjusted for age whilst the relative risks are.

^c^Likelihood ratio test statistics are calculated and p-values are obtained through Monte Carlo hypothesis testing with 9999 iterations.

^d^Areas of higher and lower than expected numbers of HIV-positive individuals.

^e^Areas of higher and lower than expected numbers of medically circumcised males aged 15–29.

aRR, relative risk adjusted for age; CI, confidence interval.

The central belt of the interpolation area was characterized as having lower HIV prevalence, with the exception of the area east of Bonda, which had high prevalence but also few data points, so estimates are less reliable. A large significant low HIV prevalence cluster was identified (overall data) covering the western-central area between Bonda and Rusape (11.3% [*n* = 1381] vs. 16.1%; aRR = 0.70, 95% CI = 0.60–0.82).

### Spatial clusters in HIV service uptake

**Figure 3.**
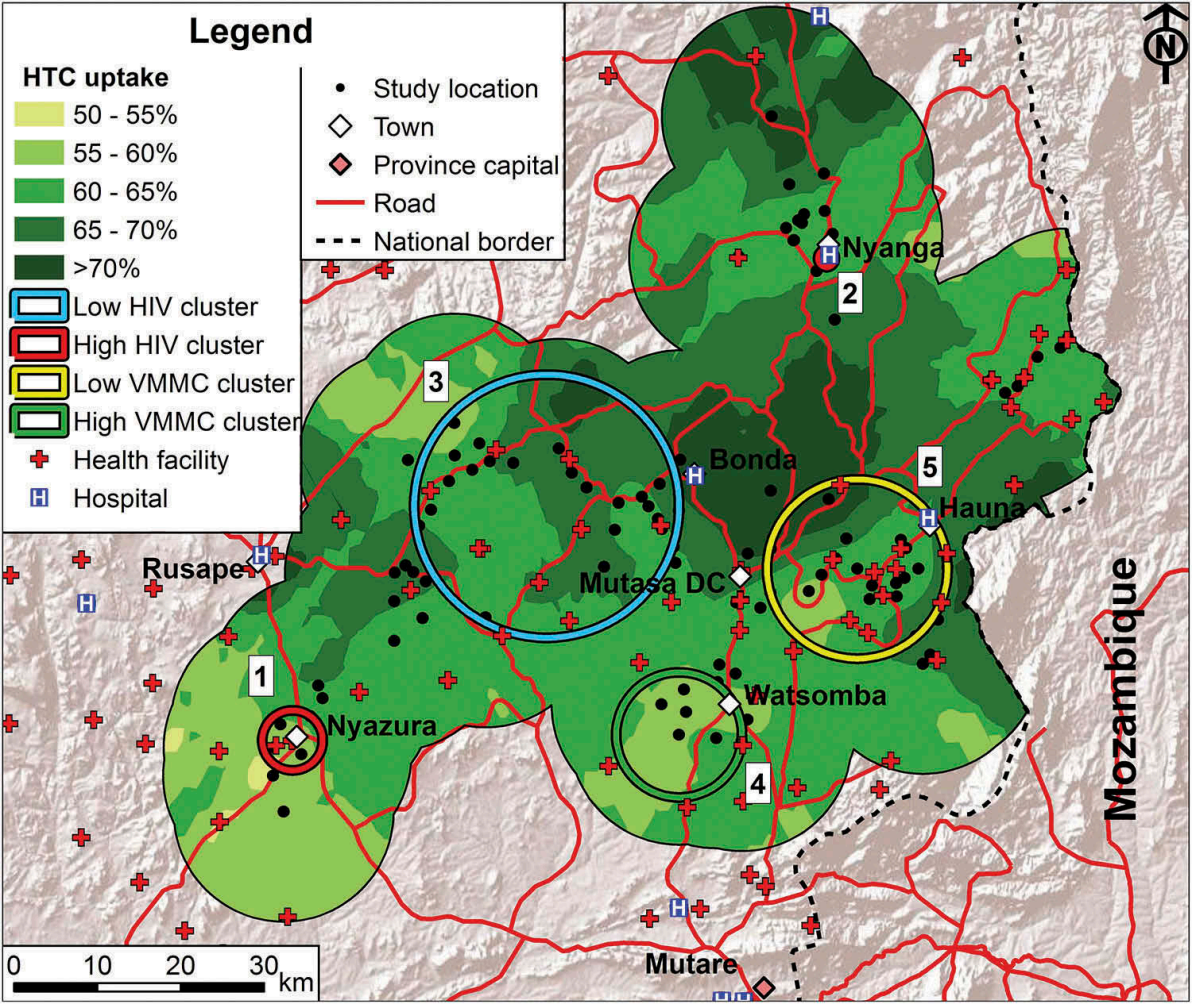
HTC and VMMC uptake in the study area in relation to health service availability. Spatial patterns of uptake of age-standardized HTC uptake (shadings on the map) and locations of VMMC uptake clusters (in the south and east) are shown in relation to the availability of health services (any type of health facility and hospitals) and the locations of the high HIV prevalence clusters (red circles around Nyazura and Nyanga) and the low HIV prevalence cluster (blue circle in the western central area) in the study area, Manicaland, Zimbabwe. The numbers next to the clusters correspond to the numbers of the clusters in Table 1.

The interpolation map for HTC uptake is presented in Figure 3; the sex-specific interpolation maps are provided in the supplementary material and show the same general patterns. The urban areas around Nyanga and Nyazura and the southern area around Watsomba tended to have lower HTC uptake than in the central area. However, no statistically significant (*p* > 0.7) clusters were found for HTC or for ART for the overall or sex-specific data (supplementary material).

Amongst males aged 15–29, one high VMMC uptake cluster near Watsomba was identified in the south of the study area (aRR = 3.26, 95% CI = 1.94–5.72) and a low VMMC uptake cluster (aRR = 0.19, 95% CI = 0.06–0.61) was found near Hauna in the east (Figure 3 and Table 1B).

### HIV service availability and uptake in the high and low HIV prevalence clusters

Figure 3 shows the patterns of HTC uptake, the clusters of high and low VMMC uptake, and the locations of hospitals and other health facilities in relation to the high and low HIV prevalence clusters. Based on self-reports, HTC and ART services were available at significantly shorter distances in the high HIV prevalence cluster but males aged 15–29 reported longer distances to the nearest place for VMMC compared to those outside the cluster (Table 2). Based on measured Euclidean distance, health facility services generally were more accessible within the cluster but hospital services were less accessible (the nearest hospital being in Rusape). Whilst there were no significant differences in uptake of VMMC and ART, HTC uptake was lower inside the high-prevalence cluster (odds of HIV test in past three years: aOR = 0.80, 95% CI = 0.66–0.97).

**Table 2.** Service availability and uptake, and socioeconomic and behavioural characteristics of people living inside the high HIV prevalence cluster around Nyazura town compared to those living in intermediate HIV prevalence areas, Manicaland, east Zimbabwe.

		Inside cluster		Inside cluster vs. outside^a^			Overall^b^	
Characteristic^c^	Units	Value	*N*	aOR	(95% CI)	*p*-Value	Value	*N*
**HIV prevalence**	(%)	25.7	494	1.98	(1.58–2.48)	<0.001	15.9	7976
**Service availability** (median distance to service providers)								
Reported distance VMMC services (males 15–29) (≤13 vs. >13 km)	km	20	53	0.20	(0.09–0.41)	<0.001	13	849
Reported distance HTC services (≤3 vs. >3 km)	km	1	469	3.60	(2.81–4.58)	<0.001	3	7334
Reported distance ART services (≤4 vs. >4 km)	km	2	269	2.70	(2.04–3.57)	<0.001	4	3434
Euclidian distance health facility (≤2.7 vs. >2.7 km)	km	0.09	517	5.13	(4.05–6.50)	<0.001	2.74	8095
Euclidian distance hospital (≤22.6 vs. >22.6 km)^d^	km	22.6	517	0.08	(0.07–0.10)	<0.001	16.7	8095
**Service uptake**								
VMMC (circumcised vs. not circumcised) (males 15–29)	%	4.50	111	1.30	(0.50–3.35)	0.586	3.76	1755
HTC uptake (HIV test in past three years vs. no HIV test in past three years)	%	58.9	497	0.80	(0.66–0.97)	0.026	63.5	7833
ART uptake (ever on ART vs. never on ART)^e^	%	52.6	97	1.34	(0.85–2.11)	0.211	50.1	1024
**Sociodemographic**								
Sex ratio (% female) (female vs. male)	%	60.1	517	1.01	(0.84–1.22)	0.91	60.7	8092
Median age (≤30 years vs. age >30 years)	Years	28	517	1.18	(0.99–1.42)	0.069	30	8095
Currently married (vs. not currently married)	%	63.5	521	1.26	(1.04–1.54)	0.020	58.9	8069
In-migration in past three years (vs. no in-migration in past three years)	%	20.3	508	1.85	(1.46–2.34)	<0.001	12.6	7986
**Socioeconomic**								
Urban or peri-urban (vs. rural)	%	100.0	517	NA^f^			31.1	8095
Primary or no education (vs. secondary or higher)	%	25.2	501	1.06	(0.85–1.33)	0.586	24.5	7870
Poorest wealth tercile (vs. intermediate or least poor)	%	26.5	517	0.49	(0.40–0.60)	<0.001	43.8	8095
**Sexual behaviour**								
Age at first sex 18 years or older (vs. ≤17 years)^g^	%	36.7	411	0.68	(0.55–0.84)	<0.001	28.2	6085
More than 1 sexual partner in past 12 months (vs. ≤ 1 partner)	%	11.2	428	1.22	(0.87–1.71)	0.242	8.7	6112
Condom use at last sex (vs. no use)	%	28.3	424	1.48	(1.18–1.86)	<0.001	21.4	6195

^a^Participants inside the high-prevalence cluster were compared to the reference group of participants outside the high- and low-prevalence clusters in the logistic regressions, adjusting for age and sex. ^b^The overall data refers to the study area as a whole, including all clusters. ^c^For the logistic regressions, the characteristic was the outcome. Continuous variables were divided along the median of the variable across the study area. The reference category is the category listed last. For example, the first service availability OR should be read as “Those inside the cluster are 0.20 times as likely to report distances to VMMC services of 13km or less”. ^d^All individuals inside the cluster have a distance to nearest hospital above the median (16.7 km), so the OR was calculated by dividing the continuous variable along the boundary of the interquartile range (22.6 km). ^e^Uptake of ART in all individuals with a positive HIV test result in the survey. ^f^All individuals inside the cluster are classified as urban/peri-urban, so no OR could be calculated. ^g^Among participants aged 18 and over. aOR, odds ratio adjusted for sex and age; CI, confidence interval.

In the low-prevalence cluster, distances to HTC services and to the nearest health facility were significantly longer compared to outside the cluster (Table 3). Hospital services, in contrast, were available at shorter distances. Nevertheless, there were no significant differences in the levels of uptake of VMMC, HTC and ART services inside and outside the low-prevalence cluster.

**Table 3.** Service availability and uptake, and socioeconomic and behavioural characteristics of people living inside the low HIV prevalence cluster between Bonda and Rusape compared to those living in intermediate HIV prevalence areas, Manicaland, east Zimbabwe.

		Inside cluster		Inside cluster vs. outside^a^			Overall^b^	
Characteristic^c^	Units	Value	*N*	aOR	(95% CI)	*p*-Value	Value	*N*
**HIV prevalence**	(%)	11.3	1381	0.62	(0.51–0.75)	<0.001	15.9	7976
**Service availability** (median distance to service providers)								
Reported distance VMMC services (males 15–29) (≤13 vs. >13 km)	km	10	143	1.16	(0.81–1.68)	0.420	13	849
Reported distance HTC services (≤3 vs. >3 km)	km	3	1306	0.83	(0.74–0.94)	0.003	3	7334
Reported distance ART services (≤4 vs. >4 km)	km	5	576	0.87	(0.72–1.04)	0.129	4	3434
Euclidian distance health facility (≤2.7 vs. >2.7 km)	km	2.8	1397	0.63	(0.56–0.71)	<0.001	2.74	8095
Euclidian distance hospital (≤16.7 vs.>16.7 km)	km	13.1	1397	1.47	(1.31–1.66)	<0.001	16.7	8095
**Service uptake**								
VMMC (circumcised vs. not circumcised) (males 15–29)	%	2.82	319	0.68	(0.33–1.39)	0.290	3.76	1755
HTC uptake (HIV test in past three years vs. no HIV test in past three years)	%	64.7	1393	1.03	(0.91–1.17)	0.664	63.5	7833
ART uptake (ever on ART vs. never on ART)^d^	%	53.7	121	1.04	(0.69–1.56)	0.869	50.1	1024
**Sociodemographic**								
Sex ratio (% female) (female vs. male)	%	63.9	1397	1.19	(1.05–1.35)	0.005	60.7	8092
Median age (≤30 years vs. age >30 years)	Years	29	1397	1.11	(0.98–1.24)	0.094	30	8095
Currently married (vs. not currently married)	%	55.7	1394	0.85	(0.75–0.97)	0.012	58.9	8069
In-migration in past three years (vs. no in-migration in past three years)	%	11.0	1391	0.85	(0.70–1.02)	0.083	12.6	7986
**Socioeconomic**								
Urban or peri-urban (vs. rural)	%	2.6	1397	0.06	(0.04–0.08)	<0.001	31.1	8095
Primary or no education (vs. secondary or higher)	%	23.8	1380	0.89	(0.76–1.03)	0.119	24.5	7870
Poorest wealth tercile (vs. intermediate or least poor)	%	57.1	1397	1.81	(1.61–2.03)	<0.001	43.8	8095
**Sexual behaviour**								
Age at first sex 18 years or older (vs. ≤17 years)^e^	%	25.4	1038	1.16	(1.00–1.36)	0.056	28.2	6085
More than 1 sexual partner in past 12 months (vs. ≤1 partner)	%	5.72	979	0.68	(0.50–0.92)	0.013	8.7	6112
Condom use at last sex (vs. no use)	%	19.4	1056	0.93	(0.78–1.10)	0.386	21.4	6195

^a^Participants inside the low-prevalence cluster were compared to the reference group of participants outside the high- and low-prevalence clusters in the logistic regressions, adjusting for age and sex.

^b^The overall data refers to the study area as a whole, including all clusters.

^c^For the logistic regressions, the characteristic was the outcome. Continuous variables were divided along the median of the variable across the study area. The reference category is the category listed last. For example, the first service availability or should be read as “Those inside the cluster are 1.16 times as likely to report distances to VMMC services of 13 km or less.”

^d^Uptake of ART in all individuals with a positive HIV test result in the survey.

^e^Amongst participants aged 18 and over.

aOR: odds ratio adjusted for sex and age; CI: confidence interval.

### Socioeconomic and sexual behavioural characteristics of populations in the high and low HIV prevalence clusters

Tables 2 and 3 compare the characteristics of the populations living inside the larger high HIV prevalence cluster around Nyazura and the low-prevalence cluster in the western-central area to those for the population living in the intermediate HIV prevalence areas not falling within the high- or low-prevalence clusters. The population living inside the high HIV prevalence cluster included more recent in-migrants, and more younger and married people than the population living in areas with intermediate prevalence. The area was more urban (100% [*n* = 517] urban or peri-urban) and the odds of being in the poorest wealth index tercile were lower inside compared to outside the clusters (aOR = 0.49, 95% CI = 0.40–0.60). There was also some evidence of higher levels of high-risk sexual behaviour within the cluster with a younger age at first sex and a non-significant higher number of sexual partners in the past 12 months. However, the odds of having used a condom during the most recent sexual intercourse were higher within the cluster (aOR = 1.48, 95% CI = 1.18–1.86).

The low HIV prevalence cluster was more rural (97.4% [*n* = 1403] classified as rural) and the odds of being in poorest wealth index tercile were higher (aOR = 1.81, 95% CI = 1.61–2.03) than in the intermediate HIV prevalence areas. The odds of being married (aOR = 0.85, 95% CI = 0.75–0.97) and of having multiple sexual partners in the past 12 months were lower in the cluster (aOR = 0.68, 95% CI = 0.50–0.92).

## Discussion

Two areas – around the small towns of Nyazura and Nyanga – in east Zimbabwe were identified as having statistically significantly higher HIV prevalence compared to the remainder of the study area, and one area – the predominantly rural western-central belt – was identified as having lower HIV prevalence, with similar patterns by sex. A socioeconomic gradient was observed with the population in the larger high HIV prevalence cluster being relatively wealthier and the population in the low HIV prevalence cluster being poorer than the population living in areas with intermediate levels of HIV prevalence. However, no differences were seen in levels of education.

No clusters of higher or lower HTC or ART uptake were identified but uptake of HTC services was reduced in the larger high HIV prevalence cluster around Nyazura despite these services being more closely available – as would be expected given the urban environment with better transport links and more health facilities. HTC and ART uptake did not appear to be reduced in the low HIV prevalence cluster despite the greater poverty and longer distances to the nearest health facilities and HTC services. Clusters of high and low VMMC uptake were identified in the south and east of the study area respectively. However, whilst VMMC services were less accessible in Nyazura than in other parts of the study area, no difference in uptake was observed. Overall, VMMC uptake was low in the study area (<4%), so population-level effects on HIV transmission are unlikely.

Whilst HTC uptake may have little impact on the behaviour of HIV-negative people [30], HTC uptake was also lower amongst HIV-positive people inside the high-prevalence cluster (supplementary material), and for these HIV testing is a prerequisite for treatment. Therefore, targeting HIV prevention resources towards areas of higher prevalence could well be cost-effective in the Nyazura area [10], particularly given the observed pattern of high-risk sexual behaviour with younger ages at first sex and a larger proportion reporting multiple sexual partners. The higher levels of condom use also fit this pattern since those who use condoms typically have more sexual partners and those who are HIV-positive report more condom use [31]. In fact, there were no differences in condom use inside and outside of the cluster in the sample of HIV-positive individuals (supplementary material). In particular, investing in more intensive promotion of HTC services, in local provision of VMMC services, and in prevention services directed towards migrants might be cost-effective. The benefits of focusing on this high-prevalence area may also extend into lower-prevalence locations given the extensive rural-urban-rural migration that occurs in Zimbabwe [32] and the links between migration and increased HIV prevalence [33]. However, whilst recent in-migrants were more common in the Nyazura high-prevalence cluster, migrant status was not a significant predictor of HIV status inside the cluster (odds of being HIV-positive: aOR = 1.55, 95% CI = 0.89–2.71, *p* = 0.12, *n* = 485, data not shown).

These findings regarding the high-risk behaviour in high HIV prevalence clusters are broadly consistent with previous studies, although there are only few studies that use spatial methods to identify high-prevalence clusters and analyse factors underlying this clustering. For example, in a study in South Africa, young women had more sexual partners inside the high-prevalence clusters [4]. Similarly, in a Ugandan study, people living in a high HIV prevalence cluster were more likely to engage in risky sexual behaviour and to have ever been married [5].

In addition to the high HIV prevalence cluster that could be targeted in a geographic approach to HIV resource allocation, a separate low VMMC uptake cluster was identified in a region with an intermediate level of HIV prevalence. A more nuanced approach to HIV resource allocation that includes other outcome measures, not only HIV prevalence, may further increase cost-effectiveness as the most appropriate interventions could be targeted to the most appropriate places.

A limitation of this study is that the data were not sampled for a spatial analysis, so the geographic spread is limited. Particularly Bayesian methods may be able to produce more precise maps of HIV prevalence and HTC [7]. Nevertheless, the combination of interpolation and cluster detection methods provides some confidence that the results reliably describe geographic variation in HIV prevalence within a generalized epidemic, as was shown in studies with similar methods [1,5]. Moreover, self-reported variables, particularly relating to sexual behaviour, may be subject to recall and social desirability bias [31], although a secret voting method was used to minimize social desirability bias for sexual behaviour variables [34]. However, these biases are likely to occur in all parts of the study area, so the comparisons of participants within and outside of the clusters should still be valid. Self-reported distance, which was used to assess availability of services, may also be imprecise, whilst Euclidian distance may not adequately reflect travel distance since roads are not considered. Although both measures gave broadly consistent results, neither measure may fully reflect actual availability of HIV services, which may be why uptake of HIV services in the rural low-prevalence cluster was not lower despite the higher travel distances. For example, the travel distance measures may not capture mobile HIV service provision and clinic characteristics in terms of services provided [26]. Limitations in the numbers of HIV-positive people in the study and statistical power may also explain why no clusters of ART uptake were detected. We may have underestimated ART uptake as CD4 count data were not available, which meant we could not account for HIV-positive people ineligible for treatment under Zimbabwe’s ART guidelines at the time of the study (initiation at CD4 ≤350 [35]); however, this underestimation is likely to occur throughout the study region, so the spatial comparisons remain valid. Finally, due to the nature of this study, a large number of statistical tests have been conducted, so some apparently significant results may have arisen by chance. Nevertheless, conclusions are supported by several variables, so there is internal consistency, and also consistency with prior hypotheses of spatial concentration of higher HIV infection risk and risky behaviour in urban areas.

This study adds to a growing body of research that demonstrates the feasibility of obtaining high-resolution geographic HIV data that would enable public health planners to use a spatial approach to resource allocation for HIV/AIDS interventions to maximize cost-effectiveness. Although it might not be feasible to obtain population-level geographic HIV data at such a high resolution for a whole country, an alternative may be to map health facilities and link antenatal surveillance data to create national maps of HIV prevalence, as has been demonstrated for Malawi [36]. Similarly, Carrel and colleagues recently demonstrated that Demographic and Health Survey (DHS) data can be used to create high-resolution HIV prevalence maps [37]. Combining different data sources, for example antenatal surveillance and DHS data, may also help to produce accurate large-scale maps [7,15]. Moreover, for Zimbabwe, the recently initiated Zimbabwe Population Based HIV Impact Assessment, covering 15,000 households across the country, may generate sufficient data density to demonstrate geographic variation in HIV prevalence nationally; similar large-scale national surveys are planned for other African countries [38].

## Conclusions

The results of this spatial analysis of a representative sample from east Zimbabwe support the targeting of geographical “hot spots” of high HIV prevalence due to extensive high-risk behaviour, lower uptake of HTC and reported poorer access to VMMC services inside the analysed high HIV prevalence cluster. Whilst infection levels in the low HIV prevalence rural communities are still substantial, the poorer access to HIV services, so far, has not resulted in disparities in uptake of VMMC, HTC or ART services in this setting. Nevertheless, if such a spatial approach to resource allocation is implemented, there are justified concerns that poorer rural populations may be further disadvantaged in terms of access to health services and that differences in uptake of HIV services may develop in the future. One possible means of avoiding this could be a general focus on the high HIV prevalence areas whilst, at the same time, increasing the provision of mobile units for HIV service delivery in less-densely populated lower HIV prevalence areas. Mobile services have been demonstrated to be effective in delivering testing and treatment services [39,40], and several studies have recently demonstrated that geographic data can support the deployment of mobile units for HIV services [41,42]. Despite the potential advantages, few health facilities in the study area currently provide mobile outreach services [26].

## Supplemental data and underlying research materials

Data produced by the Manicaland Project can be obtained from the project website: http://www.manicalandhivproject.org/data.html. Here we provide a core dataset, which contains a sample of sociodemographic, sexual behaviour and HIV testing variables from all six rounds of the main survey. If further data is required, a data request form must be completed (available to download from our website) and submitted to s.gregson@imperial.ac.uk.

## Supplementary material

To access the supplementary material to this article please see Supplementary Files under Article Tools online.
